# Dysregulation of Amino Acid Transporters in a Rat Model of TLR7-Mediated Maternal Immune Activation

**DOI:** 10.3390/pharmaceutics15071857

**Published:** 2023-07-01

**Authors:** Eliza R. McColl, Jeffrey T. Henderson, Micheline Piquette-Miller

**Affiliations:** Department of Pharmaceutical Sciences, Leslie Dan Faculty of Pharmacy, University of Toronto, 144 College St, Toronto, ON M5S 3M2, Canada; eliza.mccoll@mail.utoronto.ca (E.R.M.);

**Keywords:** placenta, pregnancy, amino acid transporters, infection, autism, neurodevelopment, neurodevelopmental disorders

## Abstract

Maternal immune activation (MIA) during pregnancy is linked to neurodevelopmental disorders in humans. Similarly, the TLR7 agonist imiquimod alters neurodevelopment in rodents. While the mechanisms underlying MIA-mediated neurodevelopmental changes are unknown, they could involve dysregulation of amino acid transporters essential for neurodevelopment. Therefore, we sought to determine the nature of such transporter changes in both imiquimod-treated rats and human placentas during infection. Pregnant rats received imiquimod on gestational day (GD)14. Transporter expression was measured in placentas and fetal brains via qPCR (GD14.5) and immunoblotting (GD16). To monitor function, fetal brain amino acid levels were measured by HPLC on GD16. Gene expression in the cortex of female fetal brains was further examined by RNAseq on GD19. In human placentas, suspected active infection was associated with decreased ASCT1 and SNAT2 protein expression. Similarly, in imiquimod-treated rats, ASCT1 and SNAT2 protein was also decreased in male placentas, while EAAT2 was decreased in female placentas. CAT3 was increased in female fetal brains. Consistent with this, imiquimod altered amino acid levels in fetal brains, while RNAseq demonstrated changes in expression of several genes implicated in autism. Thus, imiquimod alters amino acid transporter levels in pregnant rats, and similar changes occur in human placentas during active infection. This suggests that changes in expression of amino acid transporters may contribute to effects mediated by MIA toward altered neurodevelopment.

## 1. Introduction

Prenatal infections are statistically linked with increased chances of offspring being diagnosed with neurodevelopmental disorders (NDDs) including autism spectrum disorder (ASD) or schizophrenia [1]. For example, by some estimates, contracting influenza in early pregnancy can triple the chance of offspring being diagnosed with ASD [2] or schizophrenia [3]. The ongoing COVID-19 pandemic has therefore raised questions as to whether prenatal SARS-CoV-2 infection may similarly impact fetal brain development. A recent study found that babies born to people infected with SARS-CoV-2 during pregnancy were twice as likely to receive a neurodevelopmental diagnosis at one year of age [4]. Thus, the ongoing COVID-19 pandemic has emphasized the importance of deciphering the mechanisms behind prenatal infection and NDDs. 

While the exact mechanism of how prenatal infection alters neurodevelopment remains elusive, it is well established that this link occurs due to activation of the maternal immune system, a process deemed maternal immune activation (MIA), rather than the presence of a particular pathogen. This hypothesis has been strengthened by the fact that non-infectious inflammatory conditions, such as autoimmune disorders, are also associated with increased chances of offspring NDDs [5]. Moreover, pregnant rodent and non-human primate models in which MIA is induced via the toll-like receptor (TLR) agonists LPS (to model bacterial infection) or poly(I:C) (to model viral infective states) demonstrate similar neurodevelopmental features, molecular signatures, and behavioral neurobiology consistent with NDDs in humans [6]. As a result, such animal models have proven useful in order to investigate potential underlying mechanisms. The most widely utilized model for examining the impact of viral infection in mammals involves administration of poly(I:C), a synthetic, double-stranded RNA (dsRNA) molecule that stimulates TLR3. When administered at mid-gestation, poly(I:C) exposure causes a number of behavioral and neurobiological changes in offspring including microglial activation, decreased neurogenesis, altered sensorimotor gating, decreased sociability, increased repetitive behavior, and decreased working memory [7]. 

Given the critical dependence of early neural development on localized environmental factors, amino acid transporters have emerged as a possible contributor to the development of NDDs. Within the placenta, amino acid transporters mediate a net uptake of amino acids across the placenta into fetal circulation [8]. This transport is particularly important during early development before the fetus can synthesize its own amino acids, allowing for proper fetal growth [9]. Such amino acid transporters, expressed within the developing blood–brain barrier, regulate fetal brain access to amino acids requisite for proper brain development [10,11,12,13], and assist in synaptic neurotransmission of neuroactive amino acids [14]. Using the poly(I:C) MIA model, we recently demonstrated that viral MIA reduces the expression of multiple amino acid transporters in both the placenta and fetal brain [15]. In particular, we observed significantly decreased protein expression of alanine serine cysteine transporter 1 (ASCT1) and excitatory amino acid transporter 2 (EAAT2) in the placenta, and small neutral amino acid transporter 5 (SNAT5), EAAT1, and glycine transporter 1 (GLYT1) in fetal brains. Moreover, these changes in transporter expression were accompanied by functional changes in fetal brain concentrations of several amino acids, some of which are also altered in ASD and schizophrenia [15]. For this reason, we proposed that these changes in transport could contribute to the mechanism of MIA-mediated NDDs.

Given its high construct, face, and predictive validity [6], the poly(I:C) model has served as the gold standard for studying the association between viral MIA and NDDs for the past 15 years. However, many of the viruses linked to NDDs in humans (i.e., influenza, rubella, and most recently SARS-CoV-2), have single-stranded RNA (ssRNA) genomes. While TLR3 responds to dsRNA such as poly(I:C), ssRNA genomes are detected by TLR7 [16]. With this in mind, Missig et al. recently investigated the impact of prenatal TLR7 agonism on neurodevelopment using the TLR7 agonist imiquimod (IMQ), demonstrating that repeated administration of IMQ to mice during mid-gestation alters behavior, microglial activation, and the brain transcriptome of even adult offspring [17]. Interestingly, some of these findings were distinct from those observed using poly(I:C), and these changes were highly sexually dimorphic. However, it is unknown whether IMQ impacts amino acid transport in either the placenta or fetal brain. To better understand the mechanism of these effects, we therefore examined the potential role of IMQ in the dysregulation of amino acid transport as a contributing mechanism to MIA-mediated NDDs. The objective of this study was to first determine whether acute prenatal TLR7 agonism alters amino acid transport in rats. To do this, we characterized the impact of IMQ on transporter expression in the placenta and fetal brain and its effects on amino acid levels within the fetal brain. Second, we aimed to further characterize the impact of TLR7 agonism on the developing fetal brain through examination of the fetal brain transcriptome 5 days after IMQ administration. Finally, we assessed whether changes in amino acid transporter expression also occur in term human placentas during infection. 

## 2. Methods

### 2.1. Animal Experiments

Timed pregnant Sprague Dawley rats were purchased from Charles River Laboratories (Senneville, QC, Canada). Rats were maintained on a 12 h light-dark cycle with access to water and standard chow. On the morning of GD14, rats received an intraperitoneal (IP) injection of 5 mg/kg imiquimod (IMQ; HY-B0180A, MedChemExpress, Monmouth Junction, NJ, USA) dissolved in sterile, endotoxin-free water, or vehicle alone. This dose was chosen based on previous studies demonstrating its robust immune activation without impacting fetal resorption or viability [17,18,19]. The timing of IMQ administration was based on our previous study in which poly(I:C) was given on GD14 [15]. 

**Figure 1:**
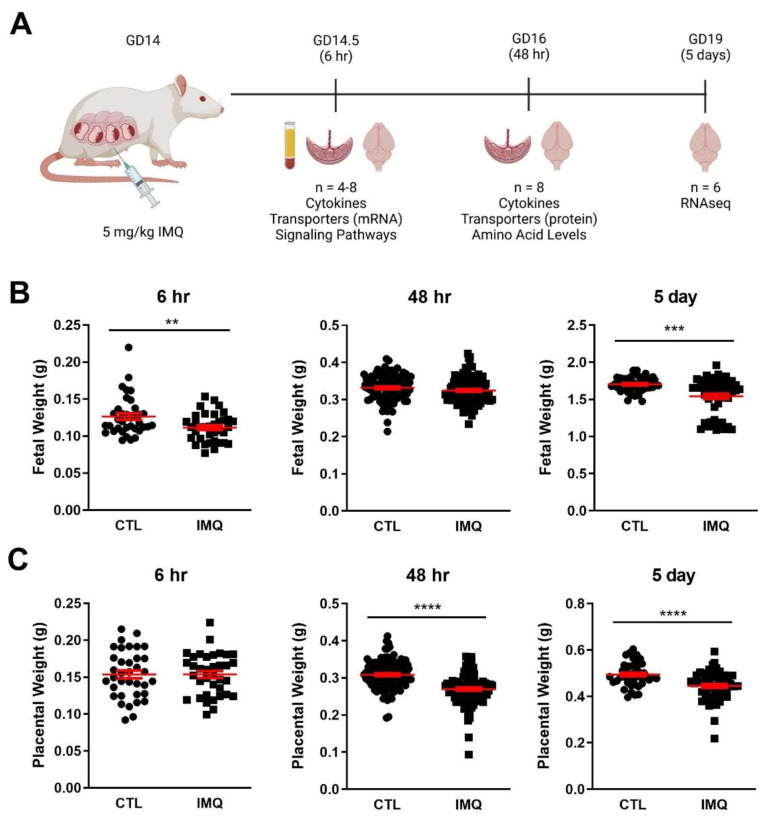
IMQ decreases fetal and placental weight. (**A**) Experimental design. Pregnant rats were given IMQ (5 mg/kg IP) on GD14 and fetal and placental tissues were collected, 6 h, 48 h, and 5 days later for subsequent analysis. (**B**) Fetal weight at multiple timepoints post-IMQ. (**C**) Placental weight at multiple timepoints post-IMQ. Significance was determined using Student’s unpaired *t*-test. ** *p* < 0.01, *** *p* < 0.001, **** *p* << 0.001. n = 36–100 fetuses/placentas (from 4–8 dams) per group per timepoint.

Dams were sacrificed 6 h (GD14.5; n = 4/group), 48 h (GD16; n = 8/group), or 5 days (GD19; n = 4/group) after IMQ injection for collection of placental and fetal tissues (Figure 1A). For the 6 and 48 h timepoints, whole fetal heads were isolated from the torso for analysis. For the 5 day timepoint, fetal brains were dissected to remove the cerebellum and brain stem to allow for collection of the neocortex and hippocampus. Tissues were snap-frozen in liquid nitrogen and stored at −80 °C for future analysis. At the time of sacrifice, maternal blood was collected in BD Vacutainer SST serum collection tubes (VWR, Mississauga, ON, Canada) and centrifuged at 1430× *g* for 15 min at 4 °C to isolate maternal serum. The serum was also snap-frozen in liquid nitrogen and stored at −80 °C. 

All animal studies were approved by the Office of Research Ethics at the University of Toronto (AUP #20011917) and conducted in accordance with the guidelines of the Canadian Council on Animal Care.

### 2.2. Sexing Rat Embryos

For 6 and 48 h timepoints, fetal sex was determined through qPCR amplification of the male-sex-determining region Y (*Sry*) in cDNA prepared from RNA extracted from corresponding fetal torsos, as described previously [15]. For the 5 day timepoint, when fetal tails were large enough to provide sufficient genetic material, fetal sex was determined through PCR amplification of *Sry* in DNA extracted from fetal tails. Fetal tails were first digested with Proteinase K overnight at 60 °C, after which potassium acetate was added to precipitate nucleic acids at −80 °C for one hour. Following centrifugation and rinsing, DNA was resuspended in nuclease-free water and subjected to PCR amplification for *Sry* and *Actb* (internal control) as visualized by gel electrophoresis as previously described [20].

### 2.3. Human Placental Sample Acquisition

Frozen human placental tissue samples were obtained from the Research Center for Women’s and Infant’s Health (RCWIH) BioBank at Mount Sinai Hospital, Toronto, Canada. Tissue samples were collected upon delivery after 37 gestational weeks from pregnancies complicated by chorioamnionitis (n = 16) or suspected infection of unknown origin (n = 6), as well as from healthy pregnancies (n = 16). For the suspected infection group, infection was not confirmed or formally diagnosed, but the individual reported cold and/or flu symptoms (i.e., fever, chills) or had an elevated white blood cell count at time of delivery. Fetal sex was not provided in clinical information supplied. All tissues were collected prior to 2016, before the beginning of the COVID-19 pandemic. 

All samples were acquired in accordance with the policies of the Mount Sinai Hospital Research Ethics Board (Protocol #12-0283-E) and followed the tenets of the Declaration of Helsinki. Sample acquisition and processing are detailed on the RCWIH BioBank website (http://biobank.lunenfeld.ca (accessed on 27 June 2023)). 

### 2.4. Quantification of Cytokines in Rat Serum

Levels of IL-6 and IL-17a in rat maternal serum were determined using ELISA kits for rat IL-6 (#R6000B, sensitivity 36 pg/mL, R&D Systems, Minneapolis, MN, USA) and rat IL-17 (#NBP1-92705, sensitivity 1.0 pg/mL, Novus Biologicals, Centennial, CO, USA) according to manufacturer’s instructions. Other immune markers were quantified in maternal serum by Eve Technologies (Calgary, AB, Canada) using a rat multiplex cytokine array.

### 2.5. RNA Extraction and qRT-PCR

mRNA levels of transporters and cytokines were quantified 6 h post-IMQ (Figure 1A) based on our previous study in which most changes at the transcript level occurred 6 h after poly(I:C)-mediated MIA [15]. RNA was extracted from rat placentas, rat fetal brains, and human placental tissue using TRIzol, as described previously [15]. Total RNA was quantified using a NanoDrop 1000 spectrometer (Thermo Fisher Scientific, Mississauga, ON, Canada). RNA was treated with DNAse1, reverse-transcribed, and then amplified and quantified using Power SYBR Green (Bio-Rad CFX384 Touch). Relative mRNA expression was determined using the comparative threshold cycle (∆∆C_t_) method and was normalized to expression of *Gapdh* in rat tissues and the mean of *YWHAZ* and *TOP1* in human placentas as internal controls. Primers for all genes analyzed are listed in Table S1. 

### 2.6. Protein Extraction

To quantify expression of membrane transporters, crude membrane protein fractions were isolated from tissues via centrifugation, as previously described [15]. Briefly, one rat placenta, one fetal brain, or 300 mg of human placental tissue were homogenized in 0.1 M Tris-HCl (pH 7.5) supplemented with protease inhibitor cocktail (#P8340, Sigma Aldrich, Oakville, ON, Canada) and PMSF. Differential centrifugation was then used to isolate a crude membrane fraction in which the relative protein content was quantified using Bradford Assay. 

For analysis of signaling proteins in cytoplasmic and nuclear fractions, a Thermo Fisher subcellular protein fractionation kit for tissues was utilized as per the manufacturer’s instructions, with the addition of PhosSTOP phosphatase inhibitor (#PHOSS-RO, Sigma Aldrich). Protein in cytoplasmic and nuclear fractions was quantified via Bradford Assay and snap-frozen in liquid nitrogen. All protein fractions were stored at −80 °C for later analysis. 

### 2.7. Western Blotting and Simple Western

Relative protein expression was quantified using either traditional Western blots or Simple Western (Protein Simple, San Jose, CA, USA). Protein expression was only assessed for transporters that have suitable commercially available antibodies and were altered at the mRNA level post-IMQ or were altered post-poly(I:C) in our previous study [15]. There is no suitable rat-specific antibody for large neutral amino acid transporter 1 (LAT1). Protein expression of transporters was assessed at 48 h post-IMQ (Figure 1A) based on our previous study in which most changes at the protein level occurred 48 h after poly(I:C)-mediated MIA [15]. In contrast, changes to signaling pathways occurred at 6 h post-poly(I:C) [15] and were therefore assessed at 6 h post-IMQ. For Simple Westerns, 1.5–3 µg of protein was separated by capillary electrophoresis under default run conditions on WES. Proteins of interest were probed using primary antibodies listed in Table S2 and detected using anti-rabbit or anti-mouse detection kits (Protein Simple). Relative protein expression was normalized to β-actin and analyzed with Compass Software version 3.1 (Protein Simple). For Western blotting, 10–50 µg of protein was separated on 10% SDS-PAGE and transferred to PVDF membranes for blocking with 5% milk in TBST. Membranes were incubated with primary antibodies listed in Table S2 overnight at 4 °C followed by washing and incubation with either HRP-conjugated anti-rabbit or anti-mouse secondary antibodies (1:75,000). Expression was normalized to β-actin (1:100,000). Bands were visualized with either SuperSignal West Femto or Dura (Thermo Fisher) and signal intensity was determined with Image Lab version 6.0.1 (Bio-Rad Laboratories Inc., Mississauga, ON, Canada)

### 2.8. Quantification of Amino Acids by HPLC

Levels of free amino acids were quantified in one male and one female fetal brain from four dams per treatment group, as well as in the corresponding maternal serum at 48 h after IMQ administration. Quantification was performed by SPARC Molecular Analysis (The Hospital for Sick Children, Toronto, ON, Canada) using reverse-phase HPLC. Detailed methods are available from the SPARC Molecular Analysis website [21]. Briefly, fetal brains were homogenized, deproteinized, and centrifuged to isolate free amino acids that were subsequently derived with phenylisothiocyanate (PITC). The resulting phenylthiocarbamyl amino acids were separated using reverse-phase HPLC and quantified with ultraviolet detection (limit of detection 50 pmoles). Quantities of each amino acid in fetal brains were normalized to the mass of brain tissue, and then to levels in maternal serum. The complete list of amino acids examined is provided in Table S3.

### 2.9. RNAseq

RNAseq was performed on RNA isolated from neonatal female cerebral cortices 5 days after IMQ administration (GD19). Total RNA was extracted using the RNeasy Plus Mini Kit (#74134, Qiagen, Germantown, MD, USA) according to the manufacturer’s instructions. Genomic DNA was depleted with DNAse1 treatment. RNA quality check, library preparation, and sequencing were performed by Princess Margaret Genomics Centre (Toronto, ON, Canada). Briefly, RNA quality was assessed using an Agilent 2100 Bioanalyzer for an RNA integrity number (RIN) greater than 8. Stranded mRNA libraries were prepared using an Illumina Stranded mRNA Prep Kit and sequenced on an Illumina Novaseq 6000 sequencer using paired-end reads (2 × 100 bp) with a read depth of 25 million. FASTQ files were quality checked using FASTQC and processed on the Galaxy platform [22]. Reads were mapped onto the rat genome (rn6) using STAR [23] and differentially expressed genes were identified with DESeq2 [24]. Enriched genes were subjected to pathway enrichment analysis with g:Profiler [25] and cross-referenced with the Simons Foundation Autism Research Initiative (SFARI) database of genes with known associations with ASD [26].

### 2.10. Statistical Analysis

With the exception of RNAseq data analysis, statistical analyses were performed using GraphPad Prism 9. Results are expressed as mean ± standard error of the mean (SEM). Vehicle and IMQ-treated rats were compared through Student’s unpaired, two-tailed *t*-tests, and human placentas were compared via Kruskal–Wallis tests with Dunn’s multiple comparisons. Significance was set to *p* < 0.05. Experimenters were not blinded to treatment conditions.

## 3. Results

### 3.1. Animal Studies: IMQ Reduces Fetal and Placental Weight

IMQ slightly but significantly reduced fetal weight by 7–9% at 6 h and 5 days (Figure 1B). Placental weight was also significantly decreased at 48 h and 5 days by approximately 7–9%. IMQ had no significant impact on litter size or number of resorptions at 48 h (Figure S1), but litter size was significantly higher than controls after 5 days (control = 10.8 ± 0.8, IMQ = 13.5 ± 0.3). 

### 3.2. IMQ Induces IL-6 in Maternal Serum and Placenta

At 6 h after IMQ administration, maternal serum levels of IL-6, IFN-γ, IP-10, MIP-1α, and MCP-1 were robustly and significantly increased (Table 1). In contrast, IL-1β, TNF-α, and IL-17a levels were not significantly affected by IMQ. 

**Figure 2:**
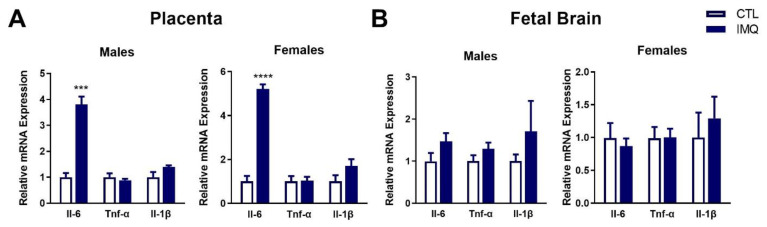
IMQ increases *Il-6* mRNA levels in fetal tissues. Transcript levels of cytokines were measured in (**A**) the placenta and (**B**) fetal brain (**B**) by qPCR 6 h after imiquimod (IMQ) administration. Expression is presented as the average ± SEM relative to controls. Significance was determined using a Student’s unpaired *t*-test. *** *p* < 0.01, **** *p* < 0.001. n = 4 per group.

In the placenta, transcript levels of *Il-6* were significantly increased in both male and female fetuses 6 h after IMQ (Figure 2A), while *Tnf-α* and *Il-1β* were not significantly affected. *Tnf-α* was significantly induced in female placentas after 48 h, with a similar trend observed in male placentas (Figure S2). In contrast, transcript levels of *Il-6*, *Tnf-α*, and *Il-1β* were not significantly altered in fetal brains at either 6 or 48 h (Figure 2B and Figure S2B).

### 3.3. IMQ Alters Signaling Pathways in Placenta

**Figure 3:**
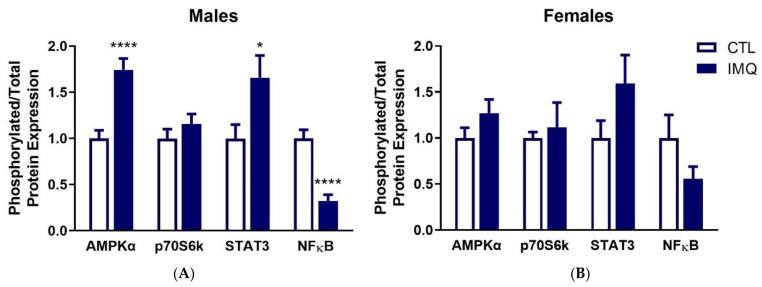
IMQ dysregulates signaling in male placentas. The ratio of phosphorylated to total protein expression for the signaling proteins indicated in (**A**) male and (**B**) female placentas was examined in IMQ-treated and control dams. Expression was assessed via Western blotting or Simple Western. Data is presented as the average ratio of phosphorylated to total protein ± SEM relative to controls. Significance was determined using a Student’s unpaired *t*-test. * *p* < 0.05, **** *p* < 0.001. n = 8 per group. Representative blots are shown in Figure S3.

The ratio of phosphorylated to total protein expression of AMPKα and the mTORC1 target p70S6k in the cytoplasm, and STAT3 and p65 of NFκB in the nucleus, was examined at 6 h after IMQ. Relative phosphorylation of AMPKα (Thr172) and STAT3 (Tyr705) was significantly increased by IMQ in male, but not female placentas (Figure 3 and Figure S3). In contrast, phosphorylation of the p65 subunit of NFκB (Ser536) was significantly decreased. While similar trends were observed for STAT3 and NFκB in female placentas, these did not reach significance. 

### 3.4. IMQ Alters Amino Acid Transporter Expression

**Figure 4:**
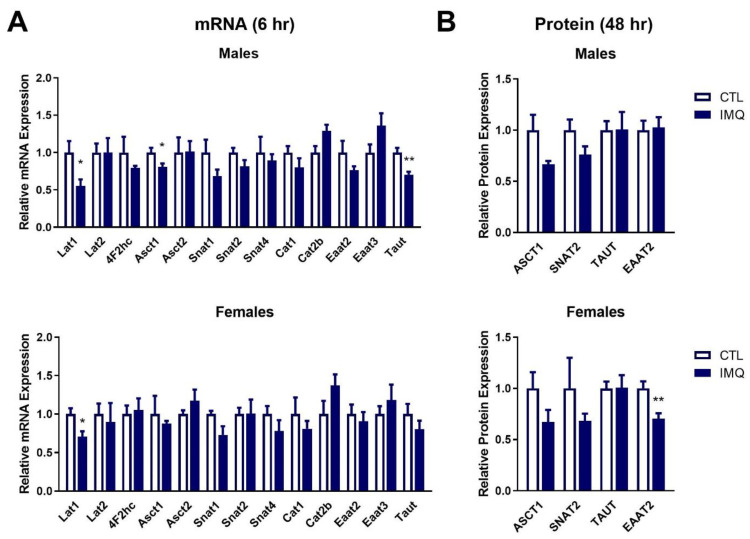
IMQ decreases amino acid transporter expression in the placenta. (**A**) mRNA and (**B**) protein expression of amino acid transporters in placentas of male and female fetuses isolated from IMQ-treated or control dams at 6 h or 48 h post-IMQ. mRNA levels were determined in samples isolated 6 h post-IMQ using qPCR, and protein expression was assessed in samples isolated 48 h post-IMQ using Western blotting. Data is presented as average expression ± SEM relative to controls. Significance was determined using Student’s unpaired *t*-test. * *p* < 0.05, ** *p* < 0.01. n = 4–8 placentas per group. Representative blots are shown in Figure S5.

In the placenta, transcript levels of *Lat1*, *Asct1*, and *Taut* were significantly decreased in males 6 h after IMQ (Figure 4A), whereas no significant changes were observed after 48 h (Figure S4A). Female placentas also exhibited decreased *Lat1* at 6 h (Figure 4A) and decreased *Taut* after 48 h (Figure S4A). At the protein level 48 h after IMQ, male placentas exhibited strong trends towards decreased ASCT1 (*p* = 0.0632) and SNAT2 (*p* = 0.0913) expression, and female placentas showed a significant decrease in EAAT2 expression (Figure 4B and Figure S5). 

**Figure 5:**
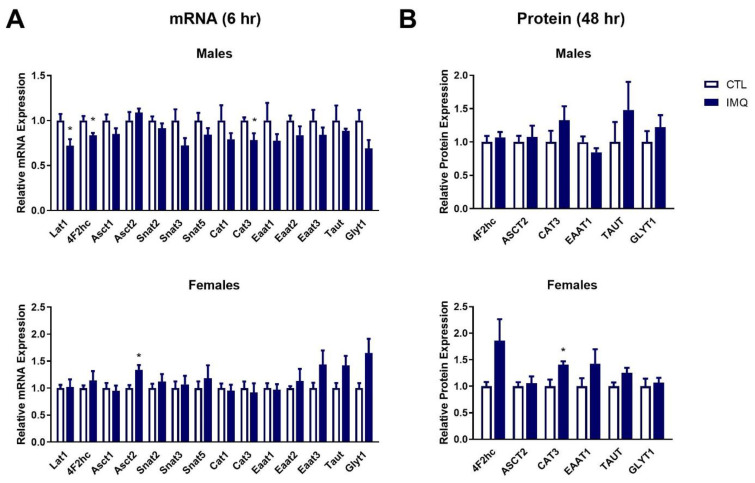
IMQ dysregulates expression of amino acid transporters in the fetal brain. (**A**) mRNA and (**B**) protein expression of amino acid transporters in the brains of male and female fetuses 6 or 48 h post-IMQ. mRNA levels were determined in samples isolated 6 h post-IMQ using qPCR, and protein expression was assessed in samples isolated 48 h post-IMQ using Western blotting. Data is presented as average expression ± SEM relative to controls. Significance was determined using Student’s unpaired *t*-test. * *p* < 0.05. n = 4–8 fetal brains per group. Representative blots are shown in Figure S6.

In the fetal brain, transcript levels of *Lat1*, *4F2hc*, and *Cat3* were significantly decreased in males 6 h after IMQ (Figure 5A). While not significant, a strong trend towards decreased *Glyt1* was also observed. In contrast, female fetal brains showed significant induction of *Asct2*, and trends towards increased *Taut* (*p* = 0.0809) and *Glyt1* (*p* = 0.0592) at 6 h. Transcript levels were unaltered in both sexes after 48 h (Figure S4B). At 48 h, CAT3 protein expression was significantly increased in females, with similar strong trends observed for TAUT (*p* = 0.0545) and 4F2hc (*p* = 0.0538) (Figure 5B and Figure S6). No significant changes in protein expression were observed in male brains. 

### 3.5. IMQ Alters Amino Acid Levels in Fetal Brains

**Figure 6:**
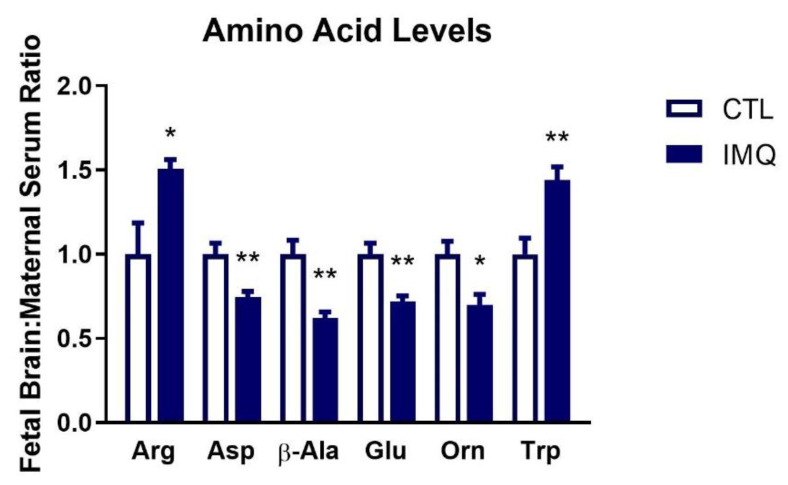
IMQ alters levels of free amino acids in fetal brains. Amino acid levels in fetal brains were quantified by HPLC 48 h after IMQ administration. The ratios of fetal brain:maternal serum concentrations are expressed as mean ± SEM, relative to controls. Significance was determined using Student’s unpaired *t*-test. * *p* < 0.05, ** *p* < 0.01. n = 8 fetal brans (4 male, 4 female) per group. Arg = arginine, Asp = aspartate, β-Ala = β-alanine, Glu = glutamate, Orn = ornithine, Trp = tryptophan.

When analyzed for males and females separately, no significant differences in the relative fetal brain to maternal serum concentrations of amino acids were observed 48 h after IMQ (Table S3). However, when males and females were combined, concentration ratios of arginine and tryptophan were significantly increased, whereas aspartate, β-alanine, glutamate, and ornithine were significantly decreased (Figure 6).

### 3.6. IMQ Alters Fetal Cerebral Cortex Expression of Genes Associated with ASD

**Figure 7:**
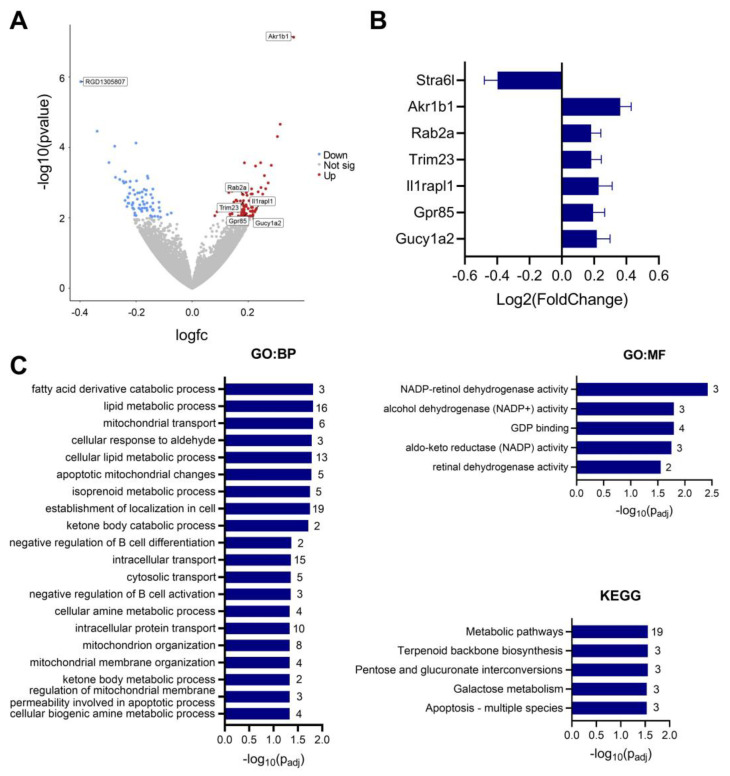
IMQ dysregulates gene expression in the female fetal cortex. Gene expression was quantified using RNAseq on brain cortices (including the hippocampus) isolated from female fetuses 5 days post-IMQ (GD19). (**A**) Volcano plot showing significantly up and downregulated genes. Genes with a *p*-value < 0.01 are highlighted red (increased) and blue (decreased). Genes of interest are labelled, with (**B**) indicating their relative Log_2_(Fold Change) ± SEM. (**C**) Pathway enrichment analysis of significantly upregulated genes from (**A**). Numbers to the right of each bar reflect the number of intersections for each pathway/process. BP = biological process, MF = molecular function. n = 6 fetal brains per group.

RNAseq of female fetal cerebral cortices revealed that the two most significantly dysregulated genes were *Akr1b1* and RGD1305807, which corresponds to *Stra6l* in the rat genome (Figure 7). An additional 165 genes were significantly dysregulated (*p* < 0.01). Pathway enrichment analysis on significantly upregulated genes revealed a number of altered metabolic processes, particularly for fatty acid metabolism (fatty acid derivative catabolic processes, lipid metabolic processes, and cellular lipid metabolic processes). Five genes from the SFARI database of genes with known associations with ASD were also significantly upregulated (*p* < 0.01): *Rab2a*, *Il1rapl1*, *Trim23*, *Gpr85*, and *Gucy1a2*. 

### 3.7. Human Placenta: Suspected Infection Decreases ASCT1 and SNAT2 Expression

**Figure 8:**
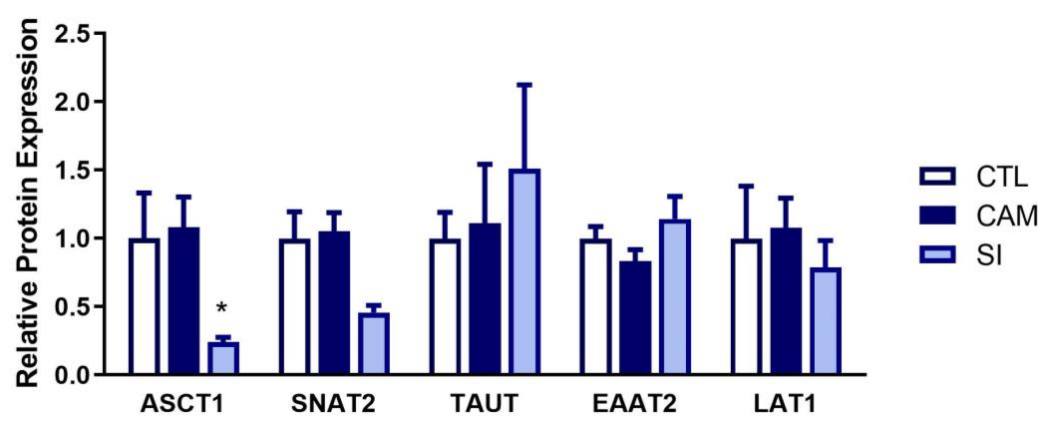
Suspected infection decreases expression of ASCT1 and SNAT2 in human placentas. Protein expression was measured in crude membrane fractions isolated from term placentas using Western blotting or Simple Western. Data is expressed relative to controls and displayed as the average ± SEM. Significance was determined using a Kruskal–Wallis test with Dunnett multiple comparison test. * *p* < 0.05. n = 16 placentas for control (CTL) and chorioamnionitis (CAM), n = 6 placentas for suspected infection (SI). Representative blots are shown in Figure S7.

In humans, ASCT1 protein expression was significantly decreased in suspected infection placentas (Figure 8 and Figure S7). SNAT2 showed a similar strong trend (*p* = 0.0872) in suspected infection, whereas EAAT2, TAUT, and LAT1 were not significantly altered in either condition. 

## 4. Discussion

There is ample evidence that viral infection during pregnancy increases the chances of offspring NDDs including ASD and schizophrenia [7]. In rodents, it is well established that prenatal TLR3 agonism with poly(I:C) alters offspring neurodevelopment. A recent report demonstrated that TLR7 agonism with IMQ also leads to changes in fetal brain development in mice [17]. However, how MIA resulting from viral infection alters neurodevelopment is less clear. We recently demonstrated that poly(I:C)-mediated MIA alters amino acid transport across the placenta and in the fetal brain of rats [15]. Given the importance of amino acids during brain development, we propose that this could be a mechanism through which MIA leads to NDDs. Here, we show that the IMQ model of MIA-mediated NDDs also triggers dysregulation of amino acid transporters in the placenta and fetal brain, alters levels of amino acids in the fetal brain, and increases expression of ASD-associated genes in the fetal cortex. Moreover, amino acid transporter expression is decreased in human placentas from pregnancies complicated by suspected infection. Together, this suggests that amino acid dysregulation is a common mechanism in models of MIA and could therefore be implicated in downstream changes in fetal brain development. 

Given that this is a relatively new model of MIA, we first sought to characterize the impact of IMQ on inflammatory markers in maternal serum, the placenta, and fetal brain. We observed a significant induction of interleukin (IL)-6 in both maternal serum and placentas of male and female fetuses 6 h after IMQ administration. Induction of IL-6 in maternal serum was also previously reported following repeated IMQ injections in pregnant mice [17]. IL-6 is known to be involved in MIA-mediated neurodevelopmental changes in mice [27,28] and elevated maternal IL-6 during pregnancy is associated with increased risk of developmental delay in humans [29]. IMQ also increased interferon gamma (IFN-γ) and the chemokines interferon gamma-induced protein (IP)-10, macrophage inflammatory protein (MIP)-1α, and monocyte chemoattractant protein (MCP)-1 in serum of IMQ-treated dams. Interestingly, all four of these markers are known to be elevated in patients with COVID-19 [30]. This is perhaps unsurprising, as like IMQ, SARS-CoV-2 is a TLR7 agonist [31]. Moreover, elevated maternal serum levels of IFN-γ at mid-gestation are associated with increased chances of ASD in human offspring [29]. Thus, the induction of the proinflammatory factors after IMQ may facilitate downstream changes in neurodevelopment. 

Next, we examined the impact of IMQ on the expression of amino acid transporters in the placenta. IMQ caused a strong trend towards reduced expression of ASCT1 in male placentas. This is consistent with our observations in the poly(I:C) model [15]. Mice lacking ASCT1/*Slc1a4* exhibit changes to behavior and brain activity [32], and gene variants are associated with intellectual disability and changes in brain morphology in humans [33]. Thus, it is possible that reduced placental ASCT1 could contribute to neurodevelopmental changes. We also observed a trend towards reduced SNAT2 protein expression in both male and female placentas. Decreased *SNAT2* mRNA has been observed in the brains of people with schizophrenia [34]. Moreover, placental SNAT2 is known to play an integral role in facilitating fetal growth; lack of placental SNAT2 causes fetal growth restriction in mice [35] and reduced SNAT2 has been observed in placentas from intrauterine growth restricted pregnancies [36]. Thus, it is possible that the reduced weight of fetuses from IMQ-treated dams could be a result of reduced SNAT2. Decreased body weight has also been observed in adult offspring of IMQ-treated pregnant mice [17], suggesting that changes in body weight may persist into adulthood. Lastly, we observed significantly reduced EAAT2 in placentas from female fetuses. This is consistent with our previous results showing that poly(I:C) reduced placental EAAT2 [15]. EAAT2 is localized to the basolateral membrane of the rat syncytiotrophoblast [37] where it helps take up glutamate from the fetal compartment which is important given that buildup of glutamate can result in neurotoxicity [38]. Thus, it is possible that the reduction in EAAT2 following IMQ may impair this functionality and impact fetal brain accumulation. 

It is plausible that IMQ-mediated effects occur through signaling pathways that are altered upon TLR7 activation. IMQ activated AMP-activated protein kinase (AMPK) in male placentas, which is consistent with what we observed following poly(I:C) [39]. Moreover, in vitro studies in placental cells demonstrated that activation of AMPK is sufficient to reduce membrane expression of ASCT1 and EAAT2 [39], suggesting that the IMQ-mediated reduction in ASCT1 and EAAT2 may be due to AMPK activation. However, EAAT2 was only decreased in female placentas, in which AMPK was not activated, so an alternative mechanism is likely responsible for this change. Inhibition of mammalian target of rapamycin complex 1 (mTORC1) has also been shown to reduce membrane expression of ASCT1, EAAT2 [39], SNAT2 [40], and LAT1 [40] in the placenta. However, IMQ did not alter phosphorylation of the mTORC1 target p70S6k in the placenta at 6 h. We observed activation of signal transducer and activator of transcription 3 (STAT3) in male placentas following IMQ, which is consistent with what has been reported in mice following poly(I:C)-mediated MIA [41]. Lastly, nuclear factor kappa B (NFκB) signaling was significantly reduced in male placentas. This could be a protective response given that NFκB activation in the placenta induces trophoblast apoptosis [42]. Aside from their impact on regulation of transporter expression, the observed changes in signaling may have additional implications for placental and fetal development. Indeed, placental mTOR and AMPK activity have been linked to altered cardiometabolic health outcomes in offspring [43]. Furthermore, both NFκB and STAT3 regulate trophoblast proliferation and invasion, suggesting that dysregulation of these pathways may impact placental development [44,45]. 

We also examined the impact of IMQ on amino acid transport in the fetal brain. At the mRNA level, IMQ-mediated changes were sexually dimorphic with males showing decreased expression of multiple transporters, whereas females exhibited increases. However, the only significant change observed at the protein level was induction of the cationic amino acid transporter 3 (CAT3) in female, but not male brains. This dysregulation could have implications for neurodevelopment, as CAT3 transports arginine [46], the precursor for nitric oxide synthesis, which is required for neuronal proliferation and differentiation [47]. Indeed, levels of arginine were significantly increased in fetal brains. A number of other amino acids were also significantly changed in fetal brains following IMQ. In addition to arginine, tryptophan was significantly increased, whereas aspartate, glutamate, ornithine, and β-alanine were significantly reduced. It is difficult to speculate as to the exact mechanism driving changes in many of these amino acids. For example, tryptophan, which was significantly increased in fetal brains, is primarily transported into the placenta by LAT1 and 2 and into the brain by LAT1 [13,48]. However, we observed a reduction in *Lat1* mRNA expression in the placenta and fetal brain, which would imply decreased rather than increased tryptophan uptake. LAT1 protein expression could not be examined in rats (there is no suitable rat-specific antibody), so whether this change in mRNA expression extends to changes in protein expression or functionality is unknown. The heterodimer of LAT1, 4F2hc, showed a trend towards increased protein expression in the fetal brain, suggesting that increased uptake may be possible; however, it is difficult to speculate without knowing LAT1 protein expression or activity. This highlights the complexity of trying to correlate specific amino acid changes with altered expression of individual transporters. Regardless of the mechanism underlying these changes, many of the affected amino acids play integral roles during neurodevelopment, and a number of the IMQ-mediated changes have been associated with NDDs. For example, increased arginine [49,50,51], decreased glutamate [52], and decreased ornithine [53,54] have been observed in the plasma or brains of individuals with ASD or schizophrenia. Moreover, higher levels of tryptophan in cord blood increase the chance of a child being diagnosed with attention-deficit/hyperactivity disorder, another NDD [55]. Furthermore, tryptophan is the precursor for the synthesis of serotonin, an essential neurotransmitter involved in neurogenesis and neuronal differentiation [56]. Thus, it is possible that these changes in amino acid levels may contribute to neurodevelopmental changes and NDDs.

Previously, IMQ-mediated MIA was shown to alter gene expression in the dorsal striatum of adult mouse offspring [17]. However, whether IMQ affects gene expression during periods of fetal brain development has not been explored. Using RNAseq, we found that prenatal IMQ exposure leads to subtle changes in the female cortical transcriptome on GD19 (5 days after IMQ). Pathway enrichment analysis of significantly upregulated genes revealed dysregulation of pathways involved in fatty acid and lipid metabolism. This is noteworthy, as altered fatty acid homeostasis and increased fatty acid metabolism have been implicated in both ASD [57] and schizophrenia [58]. Moreover, fatty acid supplementation has been shown to prevent and alleviate neurodevelopmental changes in offspring in the poly(I:C) MIA model [59,60]. The differential expression of *Akr1b1* and *Stra6l* also reflects potential changes to metabolic processes. *Stra6l* is proposed to encode an uncharacterized protein predicted to be involved in vitamin A import [61], and *Akr1b1* encodes aldose reductase, an enzyme involved in glucose metabolism. Interestingly, aldose reductase is also implicated in inflammation [62] and microglial activation [63,64]. This suggests that the induction of Akr1b1 expression following IMQ could be reflective of microglial activation in the fetal brain, which was also observed in adult mice offspring [17]. However, it is important to note that gene expression of other typical microglial activation markers (*Aif1*, *Itgam*, *Cx3cr1*, and *Tmem119*) was not significantly altered (Supplementary File S1), suggesting that microglial activation at this timepoint is unlikely.

In addition to altered expression of genes involved in metabolism, five of the upregulated genes have known associations with ASD. *Rab2a*, *Trim23*, *Gpr85*, *Gucy1a2*, and *Il1rapl1* are all listed by SFARI as candidate genes with scores of 1 (high confidence) or 2 (strong candidate) for their involvement in ASD [65,66,67,68,69]. While some of the variants in these genes that have been linked to ASD are missense mutations or protein truncations, implying reduced expression in individuals with ASD, others are associated with increased expression. For example, *Rab2a* variants linked to ASD are associated with either decreased or increased *Rab2a* gene expression. Furthermore, overexpression of *Gpr85* in mice leads to ASD-associated behavioral changes including decreased sociability, prepulse inhibition, and contextual memory [70]. Finally, loss of *Il1rapl1* in mice leads to behavioral changes opposite to those typically associated with ASD, including decreased anxiety and repetitive behavior [71]. Together, this suggests that overexpression of these genes, as was observed following IMQ, could result in ASD-like neurodevelopmental changes. 

Finally, we examined whether changes in transporter expression occur in human placentas from pregnancies complicated by either active infection or chorioamnionitis. Some of the changes observed in rats following IMQ were reflected in human placentas during suspected infection, namely a significant reduction in ASCT1 and a trend towards a 50% reduction in SNAT2. As previously mentioned, ASCT1 protein was also significantly reduced in the placenta following poly(I:C)-mediated MIA [15]. Thus, changes in transporter expression observed in rat placentas following poly(I:C) and IMQ-mediated MIA are at least partially recapitulated in human placentas during suspected active infection. In contrast, no significant changes were observed in placentas from chorioamnionitis pregnancies. One explanation for this could be that the individuals with chorioamnionitis were prescribed antibiotics earlier in pregnancy, which could have cleared the infection by the time placentas were collected and examined at term. In contrast, individuals in the suspected infection group appeared to be experiencing an active infection at the time of delivery. 

## 5. Conclusions

In conclusion, acute prenatal TLR7 agonism with IMQ in rats alters amino acid transporter expression in the placenta and fetal brain in a sex-dependent manner, and amino acid levels in fetal brains. Moreover, in females, IMQ-mediated MIA leads to altered cortical expression of genes associated with ASD. Given that results obtained in rodent models of MIA can vary depending on a number of variables including the species, strain, maternal age, type of housing, and even the manufacturer of the compound used to induce MIA [72], it will be important for future studies to examine similar outcomes to assess their robustness between studies. That being said, we also demonstrated that changes in placental transporter expression in rats are at least partially recapitulated in human placentas during suspected infection, implying that similar changes may occur in humans during MIA. In particular, we have now observed downregulation of placental ASCT1 in rats treated with poly(I:C) or IMQ, as well as human placentas during infection. While the physiological role of ASCT1 in the placenta remains unclear, ASCT1 deficiencies are associated with neurodevelopmental changes and disorders [32,33]. This suggests that the implication of MIA-mediated changes in placental ASCT1 could be an important avenue of future research.

## Figures and Tables

**Table 1:** Maternal levels of inflammation biomarkers. Concentrations of proinflammatory markers in maternal serum 6 h after administration of IMQ. Concentrations are expressed in pg/mL ± SEM. Significance was determined using an unpaired *t*-test with significance set at *p* < 0.05 (* *p* < 0.05, ** *p* < 0.01, *** *p* < 0.001).

	CTL (pg/mL)	IMQ (pg/mL)
IL-6	46 ± 12	585 ± 49 ***
IL-1β	56 ± 22	57 ± 10
TNF-α	2 ± 0.5	5.5 ± 3.5
IL-17a	0.8 ± 0.1	0.9 ± 0.2
IFN-γ	80 ± 27	361 ± 85 *
IP-10	258 ± 87	1020 ± 220 *
MCP-1	337 ± 36	1850 ± 373 **
MIP-1α	19 ± 1	44 ± 5 **

## Data Availability

The RNAseq DEseq2 output is available as a supplementary file.

## Supplementary Materials

The following supporting information can be downloaded at: https://www.mdpi.com/article/10.3390/pharmaceutics15071857/s1, Figure S1: Litter size and percentage of resorptions observed 48 h or 5 days after IMQ. Figure S2: mRNA expression of cytokines in the placenta and fetal brain 48 h after IMQ. Figure S3: Representative blots showing the effect of IMQ on signaling pathways in rat placentas. Figure S4: mRNA levels of amino acid transporters in the placenta and fetal brain 48 h post-IMQ. Figure S5: Representative blots showing the effect of IMQ on transporter expression in rat placentas. Figure S6: Representative blots showing the effect of IMQ on transporter expression in fetal rat brain. Figure S7: Representative blots showing amino acid transporter expression in human placentas at term. Table S1: Primers used for qPCR. Table S2: Antibodies used for Western blotting or Simple Western (WES). Table S3: Amino acids quantified by HPLC and separated by sex. Table S4: Summary and comparison of changes in placental transporter expression in rats and humans. File S1: DESeq2 output.
