# Supplementary material for: Dysregulation of Amino Acid Transporters in a Rat Model of TLR7-Mediated Maternal Immune Activation
Source: Pharmaceutics. 2023 Jul 1;15(7):1857. doi: 10.3390/pharmaceutics15071857 (PMC10385561; doi:10.3390/pharmaceutics15071857)
Supplement: Supplementary file 1 [file pharmaceutics-15-01857-s001.zip › Supplementary Figures and Tables.pdf]

Supplementary Data

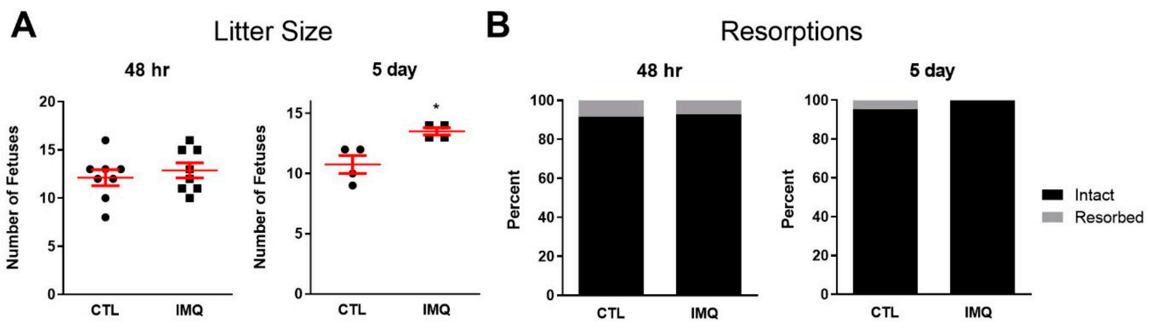

Figure S1: (A) Litter size and (B) percent of resorptions observed 48 hr or 5 days after IMQ. Significance was assessed using Student's unpaired t-test. * $p < 0.05$. n = 4 (6 hr) or 8 (5 day) litters per group.

Table S1: Primers used for qPCR. Sequences are reported 5’ to 3’.

Gene	Protein	Forward	Reverse
Rat			
Gapdh	GAPDH	GCTCTCTGCTCCTCCCTGTT	GAGGCTGGCACTGCACAA
Il-6	IL-6	GATGGATGCTTCCAAACTGGATAT	TCCAGAAGACCAGAGCAGATTTT
Tnf-α	TNF-α	GGTCCCAACAAGGAGGAGAAGT	TGGGCCATGGAACTGATGA
Il-1β	IL-1β	CGTGCTGTCTGACCCATGTG	ACTTGTTGGCCTTATGTTCTGTCCAT
Slc7a5	LAT1	CCATGATCCACCCACAGCTT	GCGTACATCAGGGTCATGACA
Slc7a8	LAT2	GGCCCTGGCTCTGATCATC	TCCAGCCAAAAGAATTCTCCTT
Slc3a2	4F2hc	ATGACAGTGAAGGGCCAAAATG	GTCACTCAGTCGCCCGGAACT
Slc1a4	ASCT1	ACGGCTTTTGCGACCTGTT	GTCCACGCCATTGTTTTCCT
Slc1a5	ASCT2	TTCCCCTCCAATCTGGTGTCT	GACAGGCACCACATGGAATG
Slc38a1	SNAT1	CGAGTGTTGGTGGTGATGGTAA	ACCCCAGGTTCTTCAAGAGACA
Slc38a2	SNAT2	CTATCTGGTCCTTCTGGTGTCCT	GTATCCTAGATTTCTCAGCAGTGACAA
Slc38a3	SNAT3	GCGGTTGCCCTGTTGTCTAG	CCCACAATCCCAGAAGACTTG
Slc38a4	SNAT4	GCTTCTTACTGTGGCAATTCTATCG	CTCCTTCCTTGGCTGTCTTCA
Slc38a5	SNAT5	CTTCACAGTTGACTCACAGATGTCTTAC	GTGCCAGGCCCCATAGC
Slc7a1	CAT1	CCGGTTCGCAGTGTGGAT	GTGCCAGGCCCCATAGC
Slc7a2	CAT2B	TGCCGTGTGCCTTGTATTACTT	CCAAGCAGACTCCTTTACTCCAA
Slc7a3	CAT3	TCGGGACTCTGCTCGCTTAC	GTCAGGCTGATATCTGAGGATAAGAA
Slc1a3	EAAT1	GCGGATGCTGCAGATGTTG	CGCCATTCCTGTGACAAGACT
Slc1a2	EAAT2	GACTGGCTGCTGGATAGAATGA	ATCCCAGCCCCAAAAGAATC
Slc1a1	EAAT3	GAGAAATTCTGATGCGGATGCT	GACACCTGTGATCATGCTGGAT
Slc6a6	TAUT	GCATCACCTGCTGGGAGAAG	GATGGACGCGTAGCCAATG
Slc6a9	GLYT1	AAAGGCGTGGGCTATGGTATG	TGCAGATGACCACGTTGTAGTAG
Sry	SRY	CCTCCAAGAACCAGAAAAGCAT	TGTTTCTGCTGTAGTGGGTATCCA
Human			
YWHAZ	14-3-3 protein zeta/delta	AGATAAAAAGAACATCCAGTCATGGA	GCCTGCTCGGCCAGTTT
TOP1	TOP1	GATGAACCTGAAGATGATGGC	TCAGCATCATCCTCATCTGC
SLC7A5	LAT1	TTCACATCCTCCAGGCTCTTCT	GAGGAGCTGTGGGTGGATCAT
SLC38A2	SNAT2	CAACAGCGACTTCAACTAC	GTAGTACCTGGATGAAATTCTG

Table S2: Antibodies used for western blotting or Simple Western (WES). R = rat, H = human

Target	Company	Catalogue Number	Dilution
AMPKα	Cell Signaling Technologies	2603	R: 1:50 (WES)<br>R: 1:1000 (Western)
phos-AMPKα (Thr172)	Cell Signaling Technologies	2535	R: 1:50 (WES)<br>R: 1:1000 (Western)
p70S6k	Cell Signaling Technologies	9202	R: 1:25 (WES)<br>R: 1:1000 (Western)
phos-p70S6k (Thr389)	Cell Signaling Technologies	9205	R: 1:500 (Western)
STAT3	Cell Signaling Technologies	4904S	R: 1:100 (WES)<br>R: 1:1000 (Western)
phos-STAT3 (Tyr705)	Cell Signaling Technologies	9145	R: 1:2000 (Western)
p65 NFκB	Cell Signaling Technologies	4764S	R: 1:100 (WES)<br>R: 1:1000 (Western)
phos-p65 NFκB (Ser536)	Cell Signaling Technologies	3033S	R: 1:1000 (Western)
ASCT1	H: Cell Signaling Technologies<br>R: Aviva Systems Biology	H: 8442<br>R: OAAN01042	H: 1:1000 (Western)<br>R: 1:300 (Western)
SNAT2	Santa Cruz Biotechnologies	sc-166366	H: 1:500 (Western)<br>R: 1:200 (Western)
TAUT	Sigma Aldrich	AB5414P	H: 1:500 (Western)<br>R: 1:500 (Western)
EAAT2	Santa Cruz Biotechnologies	sc-365634	H: 1:2000 (WES)<br>R: 1:1000 (Western)
4F2hc	Aviva Systems Biology	OAAN01460	R: 1:500 (Western)
ASCT2	Cell Signaling Technologies	5345	R: 1:200 (Western)
CAT3	Invitrogen	PIPA569257	R: 1:500 (Western)
EAAT1	Santa Cruz Biotechnologies	sc-515839	R: 1:500 (Western)
GLYT1	Aviva Systems Biology	ARP42330	R: 1:500 (Western)
LAT1	Cell Signaling Technologies	5347S	H: 1:20000 (Western)

Table S3: Amino acids quantified by HPLC and separated by sex. Amino acid levels in male and female fetal brains were quantified by HPLC 48 hr after IMQ administration. The ratios of fetal brain:maternal serum concentrations are expressed as mean ± SEM. Significance was determined within each sex using Student's unpaired t-tests with $p < 0.05$ set as significant (no significant changes were observed). n = 3-4 fetal brains per sex per group.

	**Males**		**Females**	
**Amino Acid**	**CTL**	**IMQ**	**CTL**	**IMQ**
Aspartic Acid	122.1 ± 13.8	91.4 ± 5.6	114.7 ± 9.2	84.8 ± 6.4
Glutamic Acid	110.9 ± 12.9	78.0 ± 7.0	101.9 ± 6.7	75.5 ± 3.0
Serine	14.5 ± 1.7	13.7 ± 0.9	13.3 ± 1.8	12.3 ± 0.5
Asparagine	15.4 ± 1.2	14.1 ± 1.2	13.7 ± 1.9	12.7 ± 0.9
Glycine	32.0 ± 3.5	27.3 ± 4.4	28.9 ± 1.8	26.7 ± 4.2
Glutamine	6.1 ± 0.30	5.3 ± 0.2	5.7 ± 0.55	5.0 ± 0.1
β-alanine	13.5 ± 1.0	7.6 ± 0.4	11 ± 1.5	7.4 ± 0.9
Taurine	63.1 ± 11.4	58.2 ± 4.3	56.0 ± 5.5	52.6 ± 3.6
Histidine	12.9 ± 0.7	11.4 ± 0.7	11.8 ± 1.1	10.9 ± 0.5
Arginine	21.2 ± 5.9	37.3 ± 1.8	25.4 ± 7.1	33.0 ± 0.6
Citrulline	1.4 ± 0.1	1.5 ± 0.2	1.4 ± 0.1	1.5 ± 0.2
Threonine	5.8 ± 0.7	5.5 ± 0.01	5.6 ± 0.9	5.0 ± 0.3
Alanine	9.0 ± 0.8	8.6 ± 0.2	8.8 ± 1.1	8.0 ± 0.03
Proline	7.5 ± 0.7	6.6 ± 0.5	7.0 ± 1.0	6.5 ± 0.6
Creatinine	12.6 ± 1.2	12.0 ± 0.7	11.4 ± 1.1	10.7 ± 0.8
α-Aminobutyric Acid	2.7 ± 0.3	2.2 ± 0.2	2.3 ± 0.3	2.1 ± 0.1
Tyrosine	12.1 ± 0.9	12.9 ± 0.6	10.4 ± 0.7	12.2 ± 0.4
Valine	7.2 ± 0.3	6.9 ± 0.8	6.5 ± 0.6	6.7 ± 0.6
Methionine	8.5 ± 1.0	9.2 ± 0.7	7.4 ± 0.8	8.7 ± 0.6
Isoleucine	6.3 ± 0.4	6.5 ± 0.5	5.5 ± 0.7	6.1 ± 0.4
Leucine	8.6 ± 0.4	7.6 ± 0.5	7.5 ± 0.7	7.3 ± 0.4
Phenylalanine	9.0 ± 0.5	8.4 ± 0.5	8.1 ± 0.8	8.0 ± 0.3
Tryptophan	1.2 ± 0.1	1.6 ± 0.05	1.0 ± 0.2	1.5 ± 0.1
Ornithine	12.7 ± 1.0	8.2 ± 1.2	11.1 ± 1.5	8.4 ± 1.1
Lysine	3.1 ± 0.8	4.3 ± 0.02	3.3 ± 0.6	4.2 ± 0.1

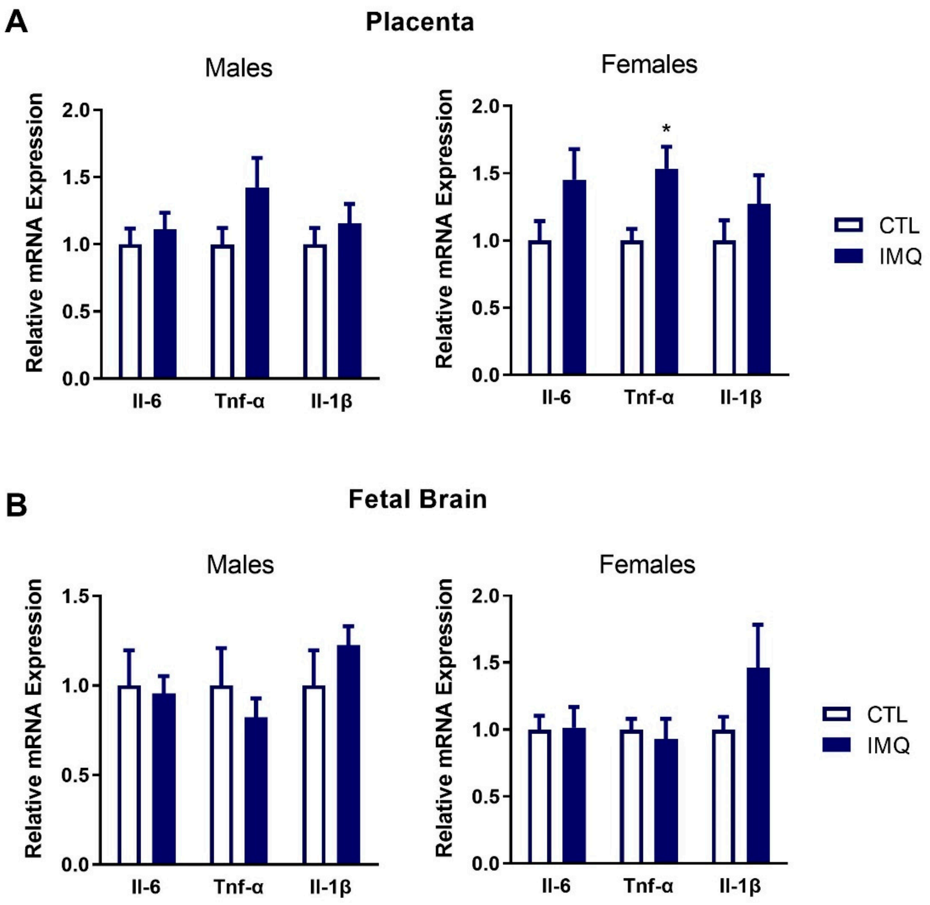

Figure S2: **mRNA expression of cytokines in (A) the placenta and (B) fetal brain 48 hrs after IMQ.** Transcript levels of cytokines were measured by qPCR. Expression is presented as the average ± SEM relative to controls. Significance was determined using a Student's unpaired t-test. * $p < 0.05$. $n = 8$ per group.

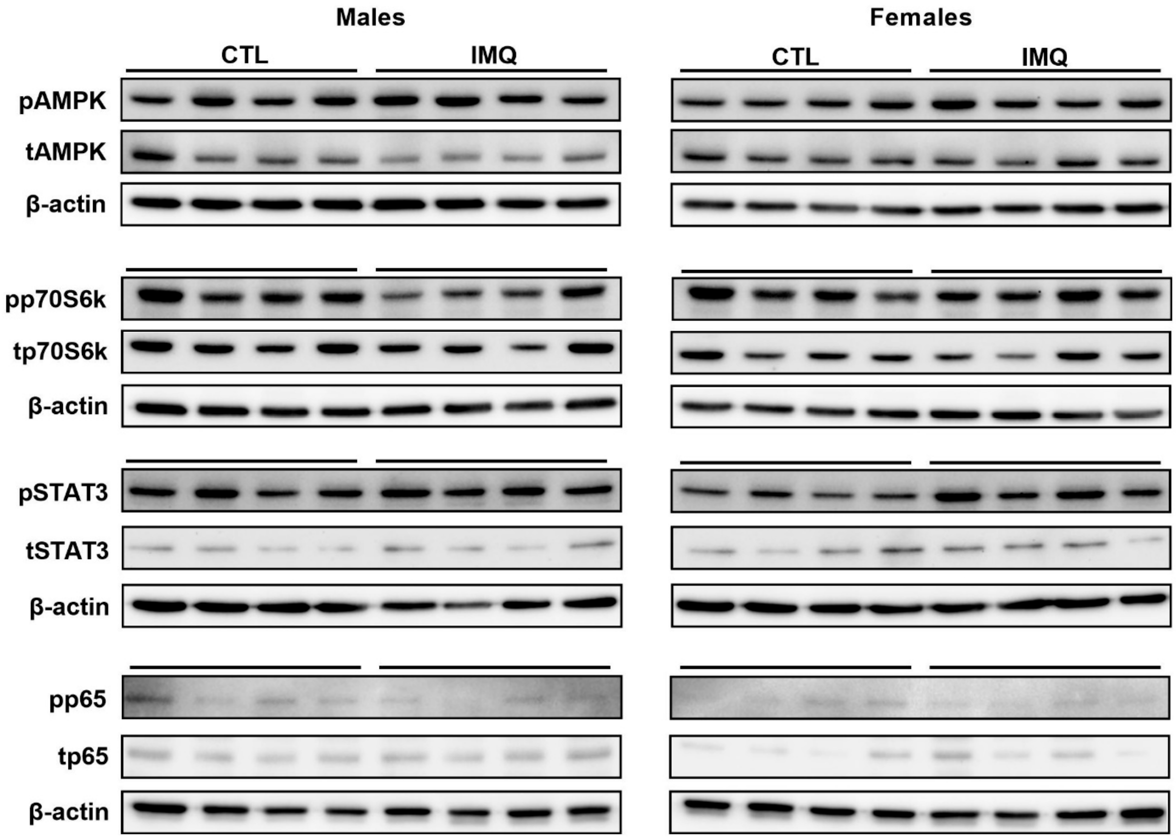

Figure S3: Representative blots showing the effect of IMQ on signaling pathways in rat placentas. Phosphorylated (p) or total (t) protein expression in male and female rat placentas was measured 6 hours after IMQ or sterile water using western blotting or Simple WES and normalized to β-actin.

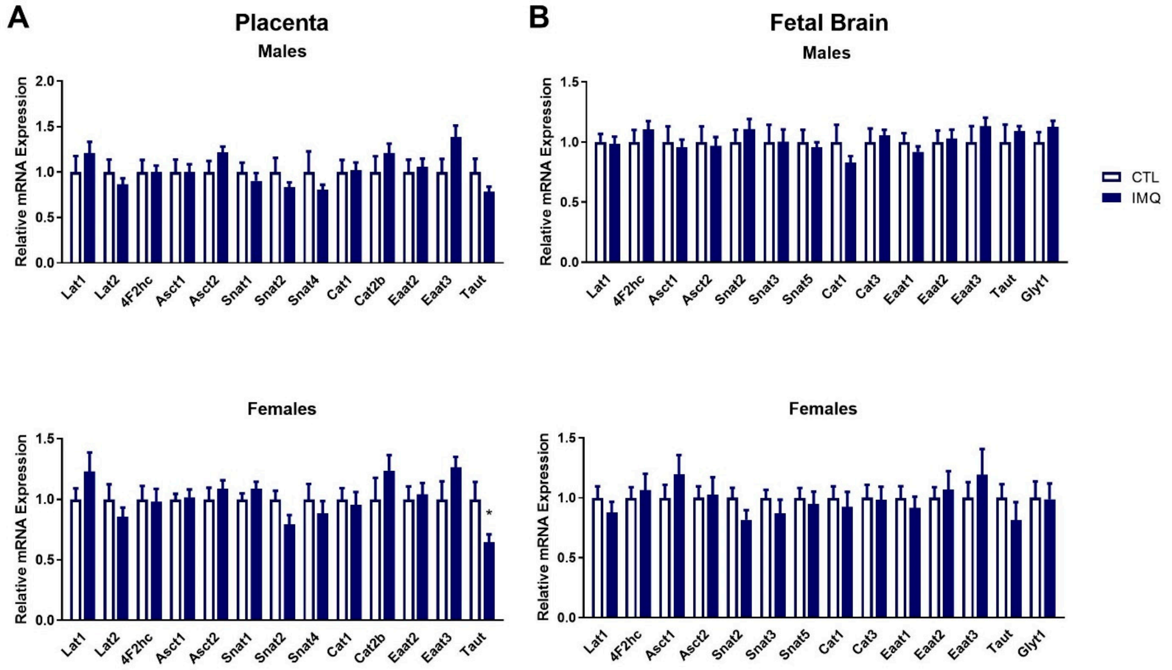

Figure S4: **mRNA levels of amino acid transporters in (A) the placenta and (B) fetal brain 48 hrs post-IMQ.** Expression was determined using qPCR and is presented as average expression ± SEM relative to controls. Significance was determined using Student's unpaired t-test. * $p < 0.05$. $n = 8$ placentas per group.

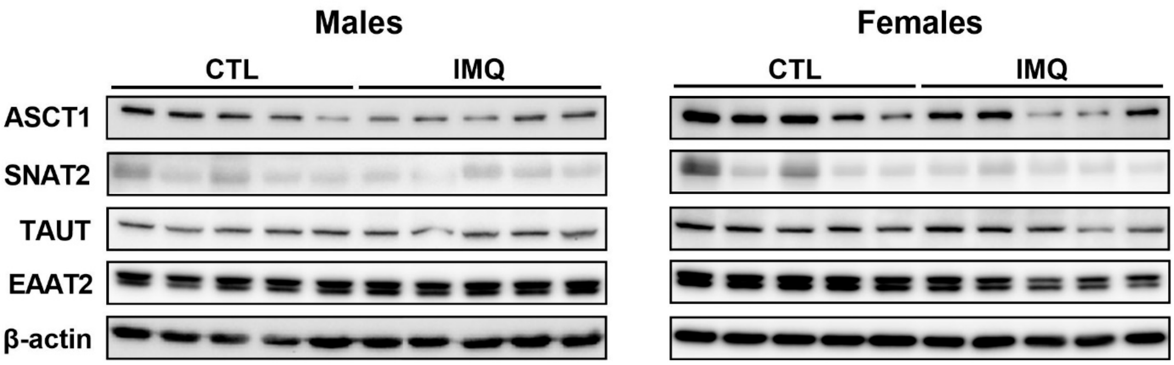

Figure S5: Representative blots showing the effect of IMQ on transporter expression in rat placentas. Protein expression of amino acid transporters in male and female rat placentas was measured 48 hours after IMQ or sterile water using western blotting or Simple WES and normalized to β-actin.

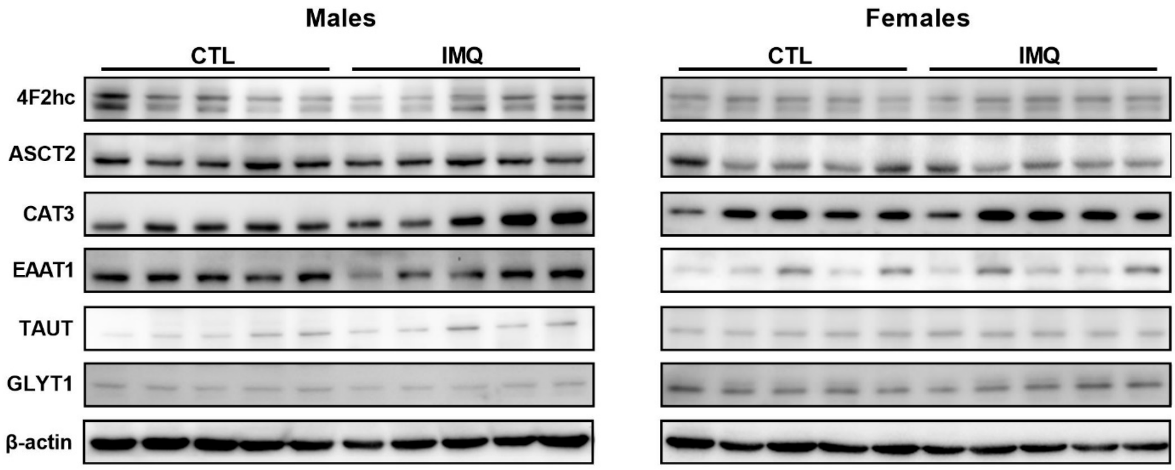

Figure S6: Representative blots showing the effect of IMQ on transporter expression in fetal rat brain. Protein expression of amino acid transporters in male and female fetal rat brains was measured 48 hours after IMQ or sterile water using western blotting or Simple WES and normalized to β-actin.

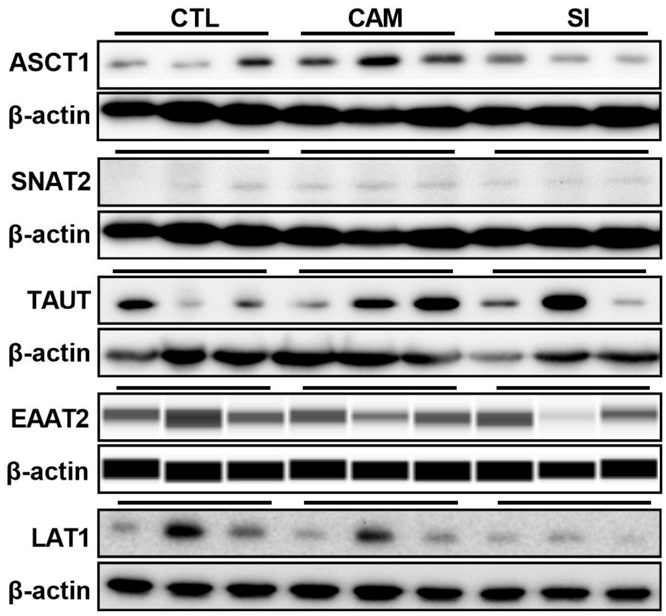

Figure S7: Representative blots showing amino acid transporter expression in human placentas at term. Protein expression of amino acid transporters was measured in human placentas complicated by chorioamnionitis (CAM) or suspected infection (SI) and compared to controls (CTL) using western blotting or Simple WES and normalized to β-actin.

Table S4: Summary and comparison of changes in placental transporter expression in rats and humans. ↓ indicates decreased expression whereas ↔ indicates no change in expression. M and F mean changes were observed in males or females, respectively. SI means that changes were observed in the suspected infection group. Unless other wise indicated by the addition of "trend", altered expression was statistically significant ($p < 0.05$).

Transporter	Rat		Human
Placenta	mRNA	Protein	Protein
LAT1	↓ (M&F)	N/A	↔
ASCT1	**↓ (M)**	**↓ (M, trend)**	**↓ (SI)**
TAUT	↓ (M)	↔	↔
EAAT2	↔	↓ (F)	↔
SNAT2	↔	**↓ (M, trend)**	**↓ (SI, trend)**
